# Targeting ferroptosis with culinary spices: a dietary strategy for neuroprotection via the Nrf2/GPX4 axis

**DOI:** 10.3389/fnut.2026.1779927

**Published:** 2026-02-12

**Authors:** Wei Hong, Yingjie Qing, Jie Liu

**Affiliations:** 1 State Key Laboratory of Technologies for Chinese Medicine Pharmaceutical Process Control and Intelligent Manufacture, Nanjing University of Chinese Medicine, Nanjing, China; 2 School of Pharmacy, Nanjing University of Chinese Medicine, Nanjing, China; 3 Department of Pharmacy, Nanjing Pukou People’s Hospital, Liangjiang Hospital, Southeast University, Nanjing, China

**Keywords:** culinary spices, ferroptosis, glutathione peroxidase 4, neurodegenerative diseases, nuclear factor-erythroid 2-related factor 2

## Abstract

The incidence of neurodegenerative diseases (NDs) continues to rise worldwide, while conventional pharmacological treatments remain limitations. Consequently, increasing attention has been directed toward to dietary intervention and nutritional supplementation as complementary strategies to prevent these diseases. Ferroptosis (an iron-dependent cell death driven by toxic lipid peroxidation) has become a key therapeutic target within NDs’ complex pathological landscape. Nuclear factor-erythroid 2-related factor 2 (Nrf2) signaling pathway is the core mechanism of neuron’s resistance to ferroptosis, which can regulate the production of the key anti-ferroptosis enzyme glutathione peroxidase 4 (GPX4). Although commonly used culinary spices are well recognized for their flavor and antioxidant effects, their specific functions as natural ferroptosis inhibitors have not been deeply studied. This review systematically evaluates the neuroprotective potential of these dietary components, and introduces in detail how they can reduce iron accumulation, lipid peroxidation, oxidative stress and neuroinflammation by activating Nrf2/GPX4 pathway. By synthesizing the current evidence, we emphasize a prospect: adding bioactive spices into our daily nutrition plan can be a promising, easy-to-obtain and safe method to help us maintain the stability of nervous system and build resistance to NDs.

## 1 Introduction

Neurodegenerative diseases (NDs), including Alzheimer’s disease (AD) and Parkinson’s disease (PD), amyotrophic lateral sclerosis (ALS) and Huntington’s disease (HD), have become more and more serious global health problems, and their characteristics are that the structure and function of neurons will gradually deteriorate (1, 2). Despite decades of research, traditional drug therapy mainly relieves symptoms and cannot stop the disease from getting worse, so now people are turning to early prevention strategies, such as adjusting diet and taking some functional food supplements (3–5). Although the etiology of NDs is complex, new research has found that ferroptosis (a programmed cell death dependent on iron) may be the key pathogenic factor (6). Different from apoptosis, ferroptosis is mainly caused by excessive iron and depletion of glutathione, which leads to the accumulation of toxic lipid peroxides (7). There are many polyunsaturated fatty acids (PUFAs) in the brain, and it is easy to accumulate iron, so neurons are naturally vulnerable to ferroptosis induced damage (8, 9). Therefore, it is a promising new direction to study how to regulate ferroptosis, which can help to protect nerve health.

The key for cells to fight against ferroptosis is the nuclear factor erythroid 2-related factor 2 (Nrf2) signaling pathway, which is the main regulator of cell protection response (10, 11). Under normal circumstances, Nrf2 will be left in the cytoplasm by Kelch-like ECH-associated protein 1 (Keap1) and then decomposed; But under stress, it will run to the nucleus to start the transcription of antioxidant genes (12). Most importantly, Nrf2 directly controls the expression of glutathione peroxidase 4 (GPX4) and cystine/glutinate antiporter (System XC-, consists of SLC7A11 and SLC3A2) (13, 14). GPX4 is recognized as the main enzyme checkpoint, which can neutralize toxic lipid peroxides, thus preventing ferroptosis (13, 15). Recent studies have shown that the down-regulation or inactivation of Nrf2/GPX4 axis is a sign of neurodegenerative diseases (16, 17). Therefore, it is a feasible therapeutic strategy to find natural compounds that can activate Nrf2 signal and restore GPX4 activity, which can enhance the ability of neurons to resist the death of ferroptosis (17, 18). Crucially, this strategy has great advantages over simple “direct” antioxidants (such as Vitamin C or E). Unlike those direct scavengers, which only neutralize ROS in a stoichiometric way and then are quickly consumed, the activation of Nrf2 will trigger the production of some enzymes, which can play a catalytic role. This brings stronger and more lasting antioxidant capacity, so that our neurons can constantly detoxify lipid peroxides without running out as quickly as direct scavengers.

The spices and condiments we usually eat not only make food more delicious, but also have great medicinal value (19). Like curcumin in turmeric, allicin in garlic etc., these natural ingredients have been found to protect our neuron system (20–22). A shared feature of these compounds is their ability to interact with Keap1, help Nrf2 to stabilize, and thus start the antioxidant system in the body (22–24). However, how these spices can prevent ferroptosis through Nrf2/GPX4 has not been completely answered. This review is to make it clear how these compounds from food sources fight ferroptosis, and let us understand why eating more spices can help prevent NDs.

## 2 Major neurodegenerative diseases

**Figure 1:**
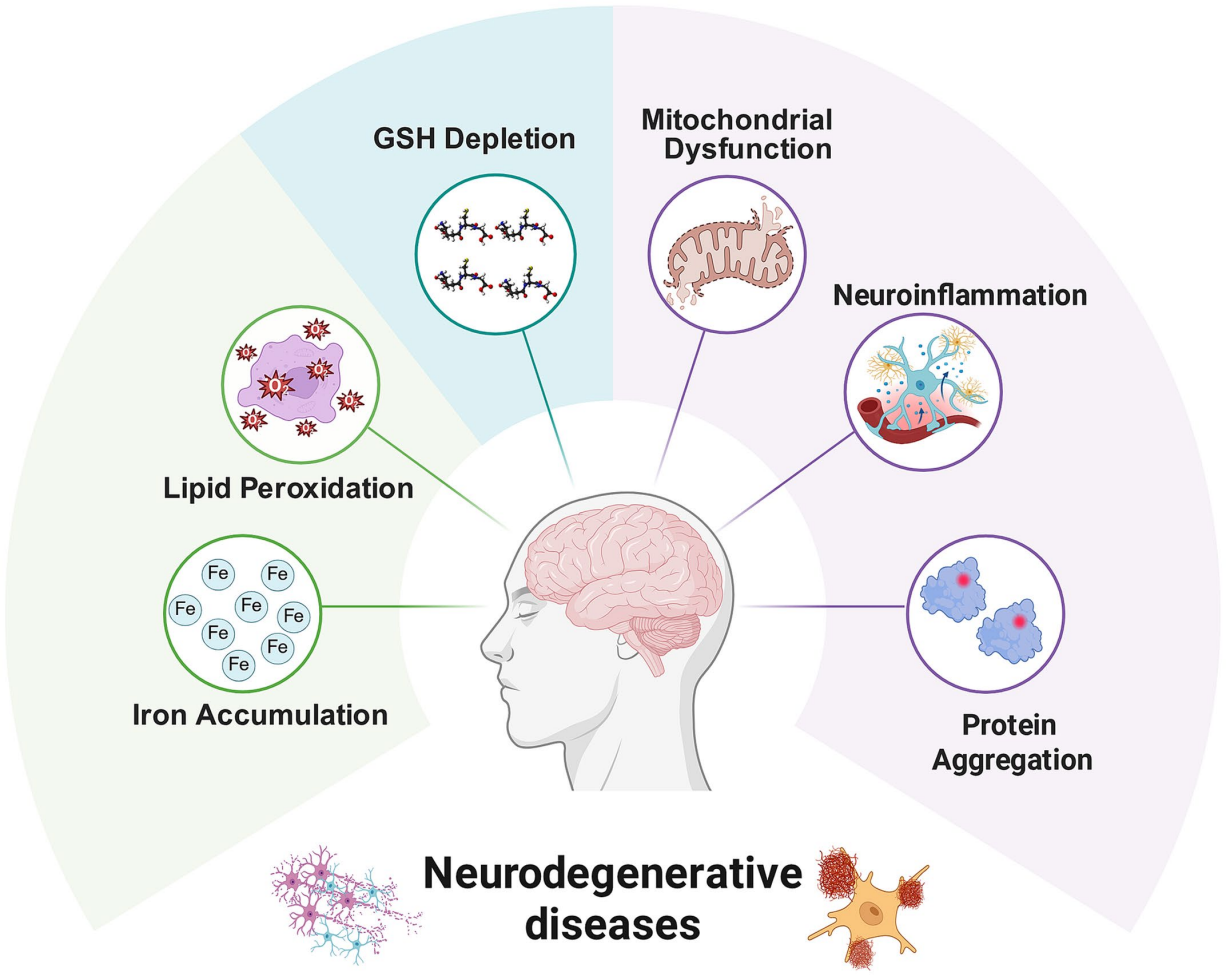
Common pathological mechanisms underlying neurodegenerative diseases.

Common pathological hallmarks shared by NDs are illustrated in Figure 1.

### 2.1 Alzheimer’s disease: the amyloid-ferroptosis loop

The AD manifests through amyloid-β (Aβ) plaque deposits and Tau protein aggregations in the brain, but new research has found that ferroptosis may be the key reason for the early neurodegeneration of AD (7, 25–27). Redox-active iron, particularly through Fe^3+^/Fe^2+^ redox cycling in complexes with amyloid-β, can promote the generation of reactive oxygen species (ROS). This process contributes to oxidative stress, accelerates Aβ aggregation, and induces neuroinflammatory responses, driving the neurotoxic processes behind AD (28, 29). Specifically, abnormal iron balance accelerates the formation of Aβ plaques and tau accumulation, exacerbating its toxic effects and promoting the development of AD (30). There is a special “Amyloid-Ferroptosis Loop” here: iron accumulation will promote the aggregation of Aβ, and these Aβ clumps will drill into the neuron membrane, producing a large number of lipid ROS, which will eventually lead to ferroptosis (31–33). In addition, the study also found that the deletion of GPX4 in the forebrain neurons of AD model exhibited neurodegeneration and behavior dysfunction, which made neurons more vulnerable to ferroptosis and oxidative damage (34, 35).

### 2.2 Parkinson’s disease: dopamine oxidation and nigral iron overload

The progress of PD is mainly due to the decrease of dopamine-producing neurons in a place called substantia nigra in the brain, the abnormal accumulation of α-synuclein (α-syn) protein, and the persistent inflammation of the nervous system. These fundamental pathological changes are all due to the fact that the redox balance in our body is broken, which leads to oxidative damage (36, 37). This disease is also related to too much iron in the body and the oxidation and destruction of lipid. Our brain is particularly vulnerable to this kind of injury, because it has a high iron content and oxidation by-products produced by dopamine metabolism (38). Studies have shown that the decrease of dopaminergic neurons is caused by ferroptosis (39).

Dopaminergic neurons are the core factory to produce dopamine, which is an unstable neurotransmitter and may induce oxidative toxicity in iron-rich substantia nigra. Unstable iron will turn dopamine into DAquinone (DAQ), which lacks electrons. Then, DAQ will form strong chemical bonds with those groups that like to donate electrons, such as those thiol groups found in hemipurine amino acids (40, 41). GPX4 is highly expressed in the brain, and some studies suggest that it may be a potential target of DAQ modification (41). In PD, downregulated System Xc- reduces cystine uptake, leading to the decrease of Glutathione (GSH), which is one of the earliest biochemical changes, directly affects the activity of GPX4 (14). If the work of GPX4 is suppressed, lipid peroxides in cells will accumulate uncontrollably. Too many of these things will destroy the cell membrane, and then start the process of cells death (42, 43).

### 2.3 Amyotrophic lateral sclerosis and Huntington’s disease

Although the symptoms of ALS and HD are very different, they both have a common problem at the molecular level, that is, mitochondrial dysfunction and lipid peroxidation (12, 44–47). In ALS, In ALS, Nrf2 cannot up-regulate System Xc-, which will lead to GSH deficiency. This will make GPX4 enzyme “hungry” and make it unable to neutralize lipid peroxidation caused by iron overload. This series of reactions directly triggered ferroptosis of motor neurons (48–51). Motor neurons are particularly sensitive to GPX4. When GPX4 does not work, it will cause nerve degeneration quickly.

The HD is a nervous system disease inherited by genes, which will make the nerve cells deteriorate (52). This disease arises from a genetic defect involving an abnormal expansion of CAG repeats within the relevant gene, which produce a harmful mutant huntingtin protein (mtHtt) (53, 54). Accumulating evidence suggests that HD progression involves a self-reinforcing pathological, in which oxidative stress, mitochondrial dysfunction, iron accumulation, lipid peroxidation, and glutathione (GSH) depletion interact and exacerbate one another, thereby contributing to progressive neuronal damage (55). The mutated huntingtin protein will disturb the Nrf2 signaling pathway, leading to the accumulation of toxic lipids (56). All these strongly indicate that ferroptosis is the main way of brain cell death when getting this disease. It is important that there is a defense mechanism centered on Nrf2 in our cells that can play a protective role. It can help to clear toxic mtHtt protein, reduce harmful brain inflammation, and restore the balance between oxidation and antioxidation in the body (57). Furthermore, Nrf2 has been shown to have anti-inflammatory effects and to regulate mitochondrial function and production (51, 58, 59). Mitochondrial dysfunction and neuroinflammation are characteristic of many neurodegenerative diseases such as ALS and HD, thus Nrf2 has become a promising therapeutic target (60).

## 3 Molecular interplay between ferroptosis and the Nrf2 signaling pathway in NDs

### 3.1 The core machinery of ferroptosis

Ferroptosis is a recently characterized, complex form of regulated cell death that is different from what we know about apoptosis, necrosis and autophagy (61). It is characterized by iron-dependent lipid peroxidation resulting from iron accumulation and the failure of cellular antioxidant systems (62). Nerve cells are particularly prone to ferroptosis because of their special cell membrane composition and energy requirements (63). There are many PUFAs on the membrane of nerve cells, which are most prone to lipid peroxidation (64–66). The specific enzymes are responsible for loading these fatty acids into phospholipids on the membrane, thus making the cell membrane sensitive to ferroptosis. While iron is indispensable for normal neuronal function, dysregulated iron homeostasis can lead to its accumulation in the brain. Excessive iron in the body is related to the development of some neurodegenerative diseases, such as AD and PD (63, 67). If there is too much iron in cells, Fenton reaction will happen. This is a catalytic process without the help of enzymes, and iron will react with hydrogen peroxide to produce ROS, particularly the highly active hydroxyl radical (68). These free radicals will attack the bis-allylic carbon atoms in PUFAs molecules on the cell membrane, and then start a self-sustaining chain reaction called lipid peroxidation. This reaction will produce toxic lipid hydroperoxides, uncontrolled accumulation of lipid hydroperoxides destroy the structure of cell membrane and mitochondrial membrane, make pores appear on the membrane, and the ions will lose balance, and ultimately triggers cell death (69).

### 3.2 System Xc- and GPX4: the primary defense checkpoints

Cells have to work normally by System Xc-/GSH/GPX4 axis, which is the most important anti-oxidation defense line in our central nervous system, to resist a severe destructive process called ferroptosis (70, 71). System Xc- is an amino acid porter which is composed of two different parts and stays on the cell membrane. System Xc- consists of SLC7A11, the substrate-specific light chain, and SLC3A2, a heavy chain subunit essential for transporter stability and membrane trafficking. It functions as a cystine–glutamate antiporter, importing cystine while exporting glutamate (72). After being brought in, cystine will soon become cysteine, and then under the action of two enzymes, Glutamate-cysteine Ligase and glutathione synthetase, Cysteine will become the key material for manufacturing GSH. GSH itself is a very important helper of “donating electrons,” which is specially used to help GPX4. GPX4 is a special selenium-containing protein, which is different from other similar enzymes, and can directly turn those complex lipid peroxides in our biofilm into non-toxic lipid alcohols. The working efficiency of GPX4 depends entirely on whether there is selenium cysteine on its active site and whether there is enough GSH in the cell. When suffering from NDs, excessive extracellular glutamate will cause excitotoxicity, which will also competitively inhibit System Xc-, thus depleting GSH in neurons, this will reduce the function of GPX4, destroy the redox balance of our cells, lead to the accumulation of iron-dependent lipid peroxides, and finally promote the cell death, making cells very fragile and prone to ferroptosis (73, 74).

### 3.3 The Nrf2 signaling pathway as a negative regulator of ferroptosis

**Figure 2:**
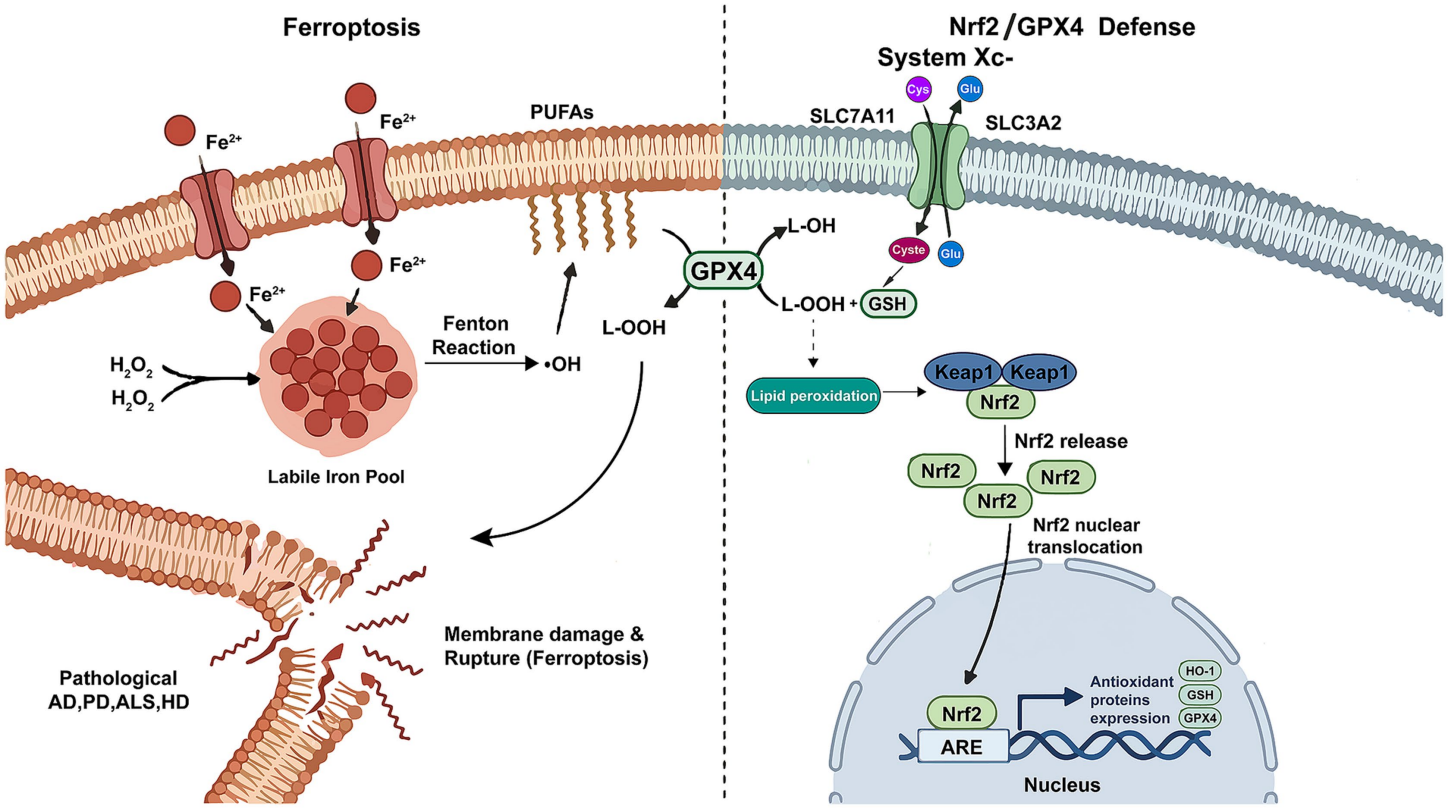
The molecular interplay between ferroptosis and the Nrf2/GPX4 antioxidant defense axis.

The antioxidant defense system in cells is mainly regulated by Nrf2, which acts as a master switch to determine whether cells can survive (50). Under normal circumstances, Nrf2 will be trapped in the cytoplasm by Keap1 and will be continuously decomposed. But Keap1 is actually a pressure sensor. When cells are exposed to oxidative stress or positively charged compounds, such as the active ingredient in spices, the specific cysteine residues on Keap1 will change. At this time, Nrf2 is released and can run into the nucleus (75, 76). Crucially, the relationship between Nrf2 and GPX4 directly regulates gene expression: the promoter region of GPX4 gene has antioxidant response elements (ARES). As soon as Nrf2 enters the nucleus, it will stick to these ARE sequences accurately, and directly let GPX4 start transcription and synthesis of protein (77, 78). In this way, the level of GPX4 is increased, and neurons can use it to destroy those toxic lipid peroxides. At the same time, Nrf2 will also increase the expression of SLC7A11, so that it can absorb more cystine to synthesize glutathione, and provide GPX4 with the helper it needs (78, 79). In addition, Nrf2 can also protect iron by increasing iron death controller ferritin heavy chain 1(FTH1), which is the main iron storage molecule in cells. Ferritin’s main function is to store iron, so that it can be dissolved without harming cells. If something goes wrong with ferritin protein, it may cause various neurodegenerative diseases (80, 81). In conclusion, activating the Nrf2 pathway is like building a comprehensive shield, which directly strengthens the defense capability of GPX4 on the one hand, and maintains the iron balance on the other hand, thus effectively stopping the ferroptosis process. The molecular interplay between ferroptosis and the Nrf2/GPX4 antioxidant defense axis is shown in Figure 2.

## 4 Nrf2 activators from culinary spices

### 4.1 Curcumin: the golden spice of neuroprotection

Curcumin, which is extracted from the rhizome of *Curcuma longa*, it is widely used as a cooking spice to add color and flavor to food. Curcumin is a core ingredient in curry and typically plays a significant role in traditional curry recipes. Curcumin gives curry its distinctive golden color and offers numerous health benefits. Beyond its role in cooking, it is also a natural compound that scientists are most concerned about when studying the activation and inhibition of ferroptosis by Nrf2 (82). There are *α*, β-unsaturated carbonyl groups in its structure, which can act as Michael receptors and react with Cys151 on Keap1 protein (83). This reaction will change the shape of Keap1, thus releasing Nrf2, which can enter the nucleus and start some genes related to antioxidation (82). In many experimental models of neurodegenerative diseases such as PD, the use of curcumin has been proved to increase the downstream antioxidant gene expression like GPX4 and heme oxygenase 1(HO-1), thus repairing the antioxidant system of GSH and reducing ROS level in cells (84–86). By maintaining the activity of GPX4, curcumin can effectively prevent the accumulation of lipid peroxide on the cell membrane of neurons and protect the integrity of mitochondria. In addition, curcumin has strong iron-chelating activity, which can catch the excess unstable iron and reduce the supply of materials for Fenton reaction, so that dopaminergic neurons and cortical neurons can be protected through a double-action mechanism to prevent them from dying because of ferroptosis (87–89).

### 4.2 Organosulfur compounds from garlic and onions

Onion vegetables that are often used as spices during cooking, especially garlic (*Allium sativum*) and onion (*Allium cepa*), are rich in organic sulfur compounds, such as allicin, diallyl sulfide (DAS) and S-allylcysteine (SAC), which have a strong anti-ferroptosis-induced NDs effect (90, 91). Different from direct antioxidants, these sulfur-containing molecules are mainly used as prodrugs and become electrophilic substances that can activate Nrf2 signal pathway after metabolism. When we chop or crush fresh garlic cloves, the enzyme alliinase in the cytoplasm will be activated, and then it will turn alliin into allicin (92). Studies have found that allicin can protect our nerves and prevent neurons from being injured by ischemia or brain injury. Moreover, it can make the nerve function better and protect the brain tissue after cerebral hemorrhage (ICH) (90, 93). It is found that arachidonate 15-lipoxygenase (ALOX15) is the key factor in the process of ferroptosis associated with ICH, and alliin can protect nerves by preventing ferroptosis mediated by ALOX15 (90). Alliin can inhibit the expression of ALOX15, which can control phospholipid peroxidation and prevent ferroptosis (90). In addition, the study found that allicin can alleviate early brain injury and protect the nervous system. It can also reduce cell death by reducing oxidative stress, inhibiting inflammatory reaction, relieving brain swelling and blood–brain barrier (BBB) damage. Together, these effects can help to improve the neurological impairment (94, 95). Therefore, allicin can enhance the ability of neurons to scavenge lipid peroxides under oxidative stress.

Moreover, aged garlic extract is an extract from kind of garlic, which contains a large number of water-soluble cysteinyl compounds, leading to its protective effects against oxidative stress and inflammation (96, 97). In addition, it may help Nrf2 enter the nucleus, so that downstream genes (such as glutamate-cysteine ligase modifier GCLM and HO-1) can remain active all the time (96). This persistent antioxidant reaction can effectively reduce the toxicity and lipid peroxidation caused by iron, indicating that eating the ingredients in allium vegetables often may be a dietary strategy and can enhance the ability of neurons to resist ferroptosis.

### 4.3 Cinnamaldehyde: the electrophilic guardian from cinnamon

Cinnamon is a natural spice obtained from the bark of *Cinnamomum* species in the Lauraceae family and widely used in culinary seasonings and traditional medicine (98). It contains the main active ingredient cinnamaldehyde, which has antioxidant, anti-inflammatory and metabolic regulating effects (99). Cinnamaldehyde is the main component that makes cinnamon have fragrance and taste (100, 101). It is a very active electron acceptor and can directly combine with Nrf2-Keap1 complex. Because it has a lively α, β-unsaturated carbonyl group, cinnamaldehyde will change the cysteine thiol group on Keap1, prevent Nrf2 from being decomposed and make it accumulate (102, 103). This will start a strong antioxidant reaction, which will increase SLC7A11 and GPX4 (104). Importantly, recent evidence shows that cinnamaldehyde exerts its anti-ferroptosis effect by lowering the level of Acyl-CoA synthetase long-chain family member 4 (ACSL4) (105). Because ACSL4 is responsible for filling the cell membrane with PUFAs (which is the main fuel of ferroptosis), inhibiting it can reduce the available raw materials for lipid peroxidation. By activating Nrf2 defense system and inhibiting pro-oxidase at the same time, cinnamaldehyde can fully protect nerve tissue and avoid ferroptosis.

### 4.4 Capsaicin and piperine: pungent defenders

Capsaicin is a spicy ingredient in *Capsicum frutescens*, and piperine is an alkaloid in *Piper nigrum* that are widely used as culinary spices to enhance flavor and appetite, which make the tongue feel prickly (106). They are also very important in regulating Nrf2/GPX4 axis (107, 108). We usually know that capsaicin is a well-known TRPV1 receptor agonist, which can lower brain oxidative stress and neuroinflammation and help to prevent neuronal death in diseases like PD, AD, cerebral stroke and epilepsy (109). A new study found that Capsaicin has its own antioxidant activity through Nrf2 (110). When using capsaicin to treat neuronal cells attacked by oxidative stressors, it can increase the accumulation of Nrf2 in the nucleus and increase the levels of HO-1 and GPX4 (110–112).

Similarly, piperine alleviated neurological defect through multiple mechanisms. In a neurotoxicity model, piperine was shown to increase the expression of Nrf2 and HO-1, thus enhancing the whole antioxidant defense system in our body (108, 113, 114). In addition, piperine can also reduce the production of iNOS, increase the levels of Nrf2 and HO-1, enhance the overall antioxidant capacity, and improve the memory function and myelin repair of hippocampus demyelinating rats (115). Interestingly, piperine can also greatly increase the absorption and bioavailability of curcumin, so if these spices are eaten together, it may have a better effect (116–118). By stabilizing Nrf2 and promoting the transcription of anti-ferroptosis related genes, these spicy ingredients help our central nervous system maintain redox homeostasis and prevent iron-dependent cell death.

## 5 Discussion

This review shows a very important new idea: it is a feasible and scientific method to deal with ferroptosis through Nrf2/GPX4, which can help us to fight NDs such as AD, PD, ALS and HD (119–122). Distinct from general oxidative stress, ferroptosis is a unique modality of iron-dependent regulated cell death driven by lethal lipid peroxidation (123). Our analysis points out that the central nervous system is particularly prone to ferroptosis because they contain a lot of PUFAs and high iron flow. Therefore, the decrease of GPX4 observed in diseases such as AD, PD and ALS is not an insignificant phenomenon, but a core cause of nerve cell membrane rupture. Therefore, therapeutic strategies should not be limited to simple free radical scavenging; rather, they should aim to restore the cellular capacity to detoxify lipid peroxides and to regulate labile iron. One of the great things about cooking spices is that they do not focus on one point like many single therapies, but they can play multiple roles at the same time. The “structure–activity relationship” of these food ingredients is very critical. Compounds such as curcumin and cinnamaldehyde have electrophilic α, β-unsaturated carbonyl groups, which enable them to covalently modify the cysteine residue on Keap1 protein like small keys. This interaction at the molecular level is equivalent to “unlocking” Nrf2, allowing it to run into the nucleus and start the transcription of such good things as System Xc- and GPX4. Importantly, these spices usually have a dual role. Curcumin, for example, is not only an activator of Nrf2, but also can directly chelate iron ions and cut off the Fenton reaction from the source (87). This multi-pronged approach can deal with the complex disease conditions than those drugs that only target a single pathway.

Moreover, there may be a synergistic effect between these spices, which means that the whole dietary intervention may protect the brain more than taking supplements alone. The problem of many plant ingredients (especially curcumin) is poor absorption, but adding piperine (from black pepper) can naturally improve the absorption rate, because it can prevent metabolic decomposition and make the ingredients eaten together more effective (116). This supports the concept of “food as medicine”, that is, eating a combined diet of turmeric, garlic, cinnamon and pepper can form a strong defense system and fight against neurodegeneration. These spices can inhibit oxidase and enhance antioxidant defense, help maintain mitochondrial health and prevent catastrophic lipid peroxidation related to NDs.

Generally speaking, it is a great and easy way to regulate Nrf2/GPX4 pathway with spices in the kitchen, which can help us to fight against the neurodegeneration caused by ferroptosis seen in diseases such as AD, PD ALS and HD. However, to turn these molecular mechanisms into effective daily eating habits, we need to use them strategically instead of eating casually. In order to maximize the benefits of protecting the brain, our diet strategy should focus on how to mix spices and eat them for a long time. For example, it is a big problem that curcumin is not easily absorbed by the body, but as emphasized in this review, eating turmeric (curcumin) and black pepper (piperine) together can make the absorption effect particularly good (117, 118). Therefore, a traditional dish like curry, which is naturally mixed with these spices, is a great example of brain-protecting diet. Similarly, taking garlic and onions as the basic ingredients for cooking every day can ensure that we can continuously obtain the organic sulfur compounds needed to replenish the GSH pool. These habits make spices no longer just condiments, but become a long-term, low-dose “health care program”, which can enhance the antioxidant protection layer of the brain. The key is to understand the therapeutic window. Because severe NDs can lead to irreversible neuronal loss, even if Nrf2 is activated, it cannot be recovered, so these dietary strategies should be mainly used as preventive measures or for prodromal stages (124, 125). Their value lies in delaying the onset and deterioration of the disease, rather than acting as a cure for advanced atrophy.

However, in order to bridge the gap between molecular mechanisms and dietary practice, we have to consider the actual situation when cooking, especially the thermal stability and food matrix effect. First of all, cooking temperature is critical to bioactivity. Alkaloids such as capsaicin is relatively heat-stable, but sulfur-containing compound in garlic are heat-sensitive (126, 127). Allicin is an activator of Nrf2, which will rapidly degraded upon exposure to high temperatures (126). Therefore, it is suggested that crushing garlic and allowing it to stand before adding it at the final stage of cooking, so as to keep its biological activity. Conversely, for lipophilic ingredients like curcumin, the cooking process plays a good role through the “food matrix effect”. Eating these spices together with fatty things (such as olive oil or milk) can not only facilitate their extraction during heating, but also stimulate bile secretion and form micelle, these processes markedly improve intestinal uptake compared with administration in aqueous forms (128, 129). Therefore, traditional cooking methods-such as using tempering spices in oil or pairing them with dietary fats actually a good method with scientific basis, which can maximize the stability and bioavailability of these neuroprotective agents.

Although promising, we still have to solve several problems to improve this method. Bioavailability is still the main bottleneck. Oral curcumin is not easily absorbed by our body, and it is metabolized quickly, so its bioavailability is particularly low, leading to its excretion rather than entry into systemic circulation (130). Future research should explore more advanced delivery systems, such as nano-formulations or lipid-based carriers, to improve the stability and brain absorption of active ingredients such as curcumin and allicin (131–133). In addition, dose standardization is also critical. Although epidemiological studies show that regular consumption is beneficial, we still lack accurate clinical guidelines to explain how much “effective dose” is needed to inhibit ferroptosis without causing gastrointestinal discomfort. Besides bioavailability and dosage, special attention should be paid to the safety of activating Nrf2. Although Nrf2 is widely recognized for its neuroprotective properties, it is often described as a ‘double-edged sword,’ as persistent activation that arising from genetic alterations can protect cancer cells from chemotherapy and facilitate tumor growth (134). However, it is important that the activation of Nrf2 caused by spices is usually transient and reversible, depending on the metabolism of those active ingredients, which is completely different from the continuous and dysregulated activation in cancer (135, 136). Nonetheless, prudence is warranted when high-dose supplementation is contemplated, particularly in older individuals who may have undiagnosed or active malignancies. In addition, patients with neurodegenerative diseases are commonly exposed to multiple medications, making potential herb–drug interactions an important practical concern. Examples include the anticoagulant effect of garlic or the inhibition of metabolic enzymes (such as CYP450) by piperine and curcumin should be considered to avoid adverse effects (137–139). Finally, strict clinical trials must be conducted to measure specific ferroptosis biomarkers in patients to confirm whether these dietary interventions can really slow down the progress of neurodegenerative diseases in clinical settings. In the final analysis, although spices cannot replace drug therapy, it can be a safe, cost-effective and multi-target adjuvant therapy by integrating them into daily diet. By creating a body environment that can resist ferroptosis through nutrition, we may be able to delay the occurrence of neurodegeneration and improve the quality of life of the elderly.

## 6 Conclusion

In conclusion, ferroptosis is the key pathogenic link of NDs, which is mainly driven by iron overload and lipid peroxidation. This review emphasizes the therapeutic potential of kitchen spices-especially curcumin, allicin, cinnamaldehyde, capsaicin and piperine-as powerful multi-target ferroptosis inhibitors. From the mechanism point of view, these bioactive compounds can provide neuroprotection by activating the classical Nrf2/GPX4 defense axis and isolating unstable iron. In addition, dietary strategies such as combining curcumin with piperine show how the synergistic combination can effectively overcome the obstacle of bioavailability. Although the future transformation research needs to solve the problems of delivery optimization and dosage standardization, integrating these spices into daily diet provides a promising, safe and economical “food-as-medicine” strategy for enhancing the resilience of neurons against neurodegeneration.

## Glossary

Aβ: amyloid-β
α-syn: α-synuclein
ARES: antioxidant response elements
AD: Alzheimer’s disease
ALS: Amyotrophic Lateral Sclerosis
ALOX15: arachidonate 15-lipoxygenase
ACSL4: Acyl-CoA synthetase long-chain family member 4
BBB: blood–brain barrier
DAS: diallyl sulfide
DAQ: DAquinone
GCLM: glutamate-cysteine ligase modifier
FTH1: ferritin heavy chain 1
GSH: Glutathione
GPX4: glutathione peroxidase 4
HO-1: heme oxygenase 1
HD: Huntington’s Disease
ICH: intracerebral hemorrhage
Keap1: Kelch-like ECH-associated protein 1
mtHtt: mutant huntingtin protein
NDs: neurodegenerative diseases
Nrf2: nuclear factor erythroid 2-related factor 2
SAC: S-allylcysteine
PD: Parkinson’s disease
PUFAs: polyunsaturated fatty acids
ROS: reactive oxygen species
